# Risk Factors of *Clostridium Difficile* Infection After Spinal Surgery: National Health Insurance Database

**DOI:** 10.1038/s41598-020-61327-1

**Published:** 2020-03-10

**Authors:** Sahyun Sung, Ji-Won Kwon, Soo-Bin Lee, Hwan-Mo Lee, Seong-Hwan Moon, Byung Ho Lee

**Affiliations:** 1 Department of Orthopedic Surgery, Severance Hospital, Yonsei University College of Medicine, Seoul, Korea; 2 Department of Orthopedic Surgery, College of Medicine, Ewha Womans University Seoul Hospital, Seoul, Korea; 3 Department of Orthopedic Surgery, National Health Insurance Corporation Ilsan Hospital, Goyang, Korea

**Keywords:** Risk factors, Clostridium difficile, Epidemiology, Orthopaedics

## Abstract

The purpose of this study was to evaluate risk factors of *Clostridium Difficile* infection (CDI) after spinal surgery using the Health Insurance Review and Assessment Service (HIRA) data. The incidence of postoperative CDI was investigated using HIRA data from 2012 to 2016. Cases involving CDI that occurred within a 30-day postoperative period were identified. Risk factors, including age, sex, comorbidities, postoperative infection, spinal surgery procedure, type of antibiotic, and duration of antibiotic use, were evaluated. Duration of hospital stay, medical cost, and mortality were also evaluated. In total, 71,322 patients were included. Presumed cases of CDI were identified in 57 patients, with CDI rate of 0.54 per 10,000 patient days. Advanced age, staged operation, postoperative infection, and the use of multiple antibiotics were significant risk factors. First-generation cephalosporins were shown to be associated with a lower incidence of CDI. CDI was also associated with longer hospital stays and increased medical cost, and it was an independent risk factor for increased mortality. Extra attention should be paid to patients at high risk for the development of postoperative CDI, and unnecessary use of multiple antibiotics should be avoided. Level of Evidence: Level III, retrospective cohort study

## Introduction

*Clostridium difficile (C. difficile)* is a common pathogen responsible for approximately 10 to 25% of all cases of antibiotic-associated diarrhea^1^. *C. difficile* infection (CDI) can occasionally lead to life-threatening complications, such as pseudomembranous colitis, intestinal perforation, and toxic megacolon^2^. The incidence of CDI is increasing globally, and more than 1.1 billion dollars have been devoted to the treatment of CDI in the United States alone^3^.

Spinal surgery is one of the most frequently performed surgeries worldwide, and the number of spinal surgeries continues to increase annually^4^, as are the rate of complex procedures and the age of spinal surgery patients^5^. Since operative mortality and morbidity increase with increasing age and surgical invasiveness, interest in the prevention of postoperative complications has grown. Several studies have reported on CDI after spinal surgery, which can be fatal^6–8^. However, because of the low incidence of CDI, previous studies focused on the incidence and demographic risk factors of patients. There has not yet been a study on the effect of medication use on the development of CDI after spinal surgery, although it is well known that some types of antibiotics and gastric acid-suppressive medications are associated with CDI.^9^

In the presence of known risk factors of CDI, the prolonged use of antibiotics and broad-spectrum antibiotics could increase the incidence of CDI. Therefore, the aim of this study was to identify the incidence of CDI after spinal surgery; risk factors thereof, including the type and duration of antibiotics use; and the effect of CDI on surgical outcomes using a large, population-based, nationally representative sample.

## Results

Of 71,322 patients, 57 were diagnosed with CDI within 30 days after surgery (incidence 0.08%). The CDI rate per total number of patient days was 0.54 per 10,000 patient days. The average time from surgery to CDI diagnosis was 18.16 ± 7.81 days. Lumbar surgery (80.68%) was the most common procedure, and the incidence of CDI therein was 0.07%. The percentage of other procedures are detailed in Table 1.

**Table 1:** Incidence of *Clostridium difficile* infection according to spinal surgery procedure.

Procedures	Total Cases, No. (%)	CDI, No.	Incidence of CDI (%)
All spine surgical procedure	71,322 (100%)	57	0.08%
Cervical	9,179 (12.87%)	10	0.11%
Thoracic	1,282 (1.80%)	1	0.08%
Lumbar	59,541 (83.48%)	44	0.07%
Multilevel	1,320 (1.85%)	2	0.15%

CDI: *Clostridium Difficile* infection.

Multilevel indicates the simultaneous operation of two or more of the cervical, thoracic, and lumbar regions.

Patients who acquired postoperative CDI after spinal surgery were significantly older (67.12 ± 10.94 vs. 57.27 ± 14.78); there was no difference in sex (Table 2). The proportion of patients over 65 years of age was greater in the CDI group than in the non-CDI group (P < 0.001). CDI patients also had a higher prevalence of chronic obstructive pulmonary disease (P = 0.030) and a higher incidence of postoperative infection, such as urinary tract infection (P < 0.001), sepsis (P < 0.001), or pneumonia (P < 0.001), compared to patients without CDI. Patients with CDI were more likely to have been prescribed proton pump inhibitors postoperatively (P = 0.002); however, there was no significant difference in the use of H2 blockers between the CDI and non-CDI groups. As for the use of antibiotics, broad spectrum antibiotics were more often prescribed to CDI patients, including third generation cephalosporins (P < 0.001), fourth generation cephalosporins (P = 0.013), penicillins (P < 0.001), glycopeptides (P < 0.001), carbapenems (P = 0.009), and ketolides (P = 0.043). On the other hand, CDI patients were less likely to be given first generation cephalosporins (P = 0.004).

**Table 2:** Patient characteristics, comorbidities, and prescribed medications in, with, and without *Clostridium difficile* infection.

Population	CDI (57)	No CDI (71,322)	P Value
Mean age	67.12 ± 10.94	57.27 ± 14.78	<0.001
Age groups, years (%)			<0.001
0–45	3.51%	21.50%	
46–65	38.60%	45.41%	
>65	57.89%	33.09%	
Sex			0.155
Female	57.9%	48.5%	
Male	42.1%	51.5%	
Baseline comorbidities			
Hypertension	45.61%	37.95%	0.234
Diabetes mellitus	28.07%	19.49%	0.102
COPD	8.77%	3.06%	0.030
Stage operation	5.26%	0.24%	<0.001
Postoperative infection			
Urinary tract infection	19.30%	2.37%	<0.001
Sepsis	33.33%	6.82%	<0.001
Pneumonia	19.30%	1.23%	<0.001
Surgical site infection	0.03%	1.75%	0.016
Any infection	61.40%	25.27%	<0.001
Additional postoperative prescription			
Proton pump inhibitor	29.82%	15.07%	0.002
H2 blocker	89.46%	81.87%	0.136
Lactobacillus	3.51%	1.50%	0.211
Antibiotic types			
1^st^ generation cephalosporins	52.63%	70.26%	0.004
2^nd^ generation cephalosporins	49.12%	50.94%	0.784
3^rd^ generation cephalosporins	40.35%	15.86%	<0.001
4^th^ generation cephalosporins	3.51%	0.30%	0.013
Aminoglycosides	17.54%	12.67%	0.269
Penicillins	22.81%	8.17%	<0.001
Quinolones	21.05%	13.32%	0.086
Glycopeptides	22.81%	4.13%	<0.001
Carbapenems	5.26%	0.75%	0.009
Ketolides	5.26%	1.37%	0.043
Others	12.28%	1.80%	<0.001

CDI: *Clostridium difficile* infection; COPD: Chronic obstructive pulmonary disease.

The effect of the duration of antibiotics use and the number of co-administered antibiotics on the incidence of CDI was investigated. (Table 3) The higher the number of administered antibiotics, the higher the incidence of CDI (P < 0.001). There was no significant difference according to the duration of antibiotics used.

**Table 3:** *Clostridium difficile* infection incidence according to the duration of antibiotic use and the number of administered antibiotics.

	Total Cases, No. (%)	CDI, No.	Incidence of CDI (%)	P Value
^†^Duration of antibiotic use (day)				0.201
7 or less	44802 (62.8%)	31	0.07	
8 ~ 14	21709 (30.4%)	21	0.10	
15 or more	4811 (6.7%)	5	0.10	
Number of antibiotics				<0.001
1	32047 (44.9%)	14	0.04	
2	26122 (36.7%)	19	0.07	
3 or more	13123 (18.4%)	24	0.18	

CDI: *Clostridium difficile* infection ^†^The duration of antibiotic use was calculated as the days which the antibiotics were administered, regardless of the number of antibiotics or the route of administration.

In multivariate analysis, independent preoperative/postoperative risk factors for postoperative CDI included greater age (most notably ≥65 years old, OR = 6.408, 95% confidence interval (CI) = 1.521–26.997, P = 0.011), staged operation (OR = 5.336, 95% CI = 1.417–20.087, P = 0.013), postoperative infection such as urinary tract infection (OR = 3.538, 95% CI = 1.698–7.371, P = 0.001), sepsis (OR = 4.427, 95% CI = 2.458–7.975, P < 0.001), and pneumonia (OR = 6.096, 95% CI = 2.793–13.303, P < 0.001) (Table 4). First-generation cephalosporins (OR = 0.408, 95% CI = 0.226–0.738, P = 0.003) were associated with a lower incidence of CDI. The use of three or more antibiotics was associated with higher CDI incidence (OR = 2.808, 95% CI = 1.266–6.229, P = 0.011). Third- and fourth-generation cephalosporins, penicillins, glycopeptides, carbapenems, and ketolides, which previously were shown to be associated with increased incidence of CDI, were not identified as significant risk factors for CDI in the multivariate model. The duration of antibiotics used also did not have a significant effect.

**Table 4:** Independent risk factors for postoperative *Clostridium difficile* infection.

Risk Factor	Odds ratio	Lower 95% CI	Higher 95% CI	*P value
Age, years				
≤45	Ref.			
45–65	4.526	1.061	19.312	0.041*
>65	6.408	1.521	26.997	0.011*
Staged Operation	5.336	1.417	20.087	0.013*
Postoperative infection				
Any infection	3.215	1.849	5.589	<0.001*
UTI	3.538	1.698	7.371	0.001*
Sepsis	4.427	2.458	7.975	<0.001*
Pneumonia	6.096	2.793	13.303	<0.001*
Type of Antibiotics				
1^st^ generation Cephalosporin	0.408	0.226	0.738	0.003*
2^nd^ generation Cephalosporin	0.600	0.328	1.095	0.096
Quinolone	0.379	0.180	0.796	0.010*
Number of Antibiotics				
1	Ref.			
2	2.010	0.976	4.140	0.058
3 or more	2.808	1.266	6.229	0.011*

CI: Confidence Intervals; UTI: Urinary tract infection; SSI: Surgical site infection.

*Significant P values < 0.05.

*R*^2^ = *0.159*.

Postoperative CDI after spinal surgery resulted in remarkably negative outcomes. Mean hospitalization period increased more than three-fold for patients diagnosed with CDI versus. those not diagnosed with CDI (64.91 ± 74.87 days vs. 18.85 ± 25.37 days, P < 0.001). When multiple regression analysis was performed to investigate risk factors for increased hospitalization period, CDI increased the hospitalization period increased by 30.12 days (P < 0.001) after adjusting for other variables. The total medical cost increased by more than four-fold for those patients with CDI diagnosis ($14,704 ± 15,590 vs. $3,580 ± 4,190, P < 0.001).

Multivariate analyses, identifying independent risk factors for mortality within 30 days postoperatively, identified CDI as a significant risk factor (OR = 7.717, 95% CI = 1.670–35.662, P = 0.009) (Table 5). In addition, the use of antibiotics, such as fourth-generation cephalosporins, penicillins, glycopeptides, and carbapenems, were associated with significantly increased odds of death, whereas the first- and second-generation cephalosporins were associated with lower risk. Advanced age and the use of three or more antibiotics were also associated with an increased odds of death.

**Table 5:** Independent risk factors for postoperative mortality within 30 days after surgery.

Risk Factor	Odds ratio	Lower 95% CI	Higher 95% CI	P value
CDI	7.717	1.670	35.662	0.009*
Age, years				
≤45	Ref.			
45–65	1.925	1.008	3.677	0.047*
>65	2.809	1.489	5.299	0.001*
Postoperative infection				
Any infection excluding CDI	0.660	0.440	0.988	0.044*
Pneumonia	2.939	1.786	4.837	<0.001*
Type of Antibiotics				
1^st^ generation cephalosporins	0.638	0.424	0.959	0.031*
2^nd^ generation cephalosporins	0.597	0.394	0.904	0.015*
4^th^ generation cephalosporins	2.910	1.483	5.707	0.002*
Penicillins	2.813	1.838	4.306	<0.001*
Glycopeptides	2.051	1.288	3.267	0.002*
Cabapenems	1.878	1.089	3.238	0.023*
Number of antibiotics				
1	Ref.			
2	1.458	0.816	2.604	0.202
3 or more	3.016	1.648	5.519	<0.001*

CI: Confidence Intervals; CDI: Clostridium Difficile infection.

*Significant P values < 0.05.

*R*^2^ = *0.284*.

## Discussion

In this study, we found CDI after spinal surgery to be associated with advanced age, staged operation, postoperative infection, and administration of three or more antibiotics. The third-generation cephalosporins, clindamycins, penicillins, and fluoroquinolones were previously reported as risk factors for CDI^10,11^. In this study, first-generation cephalosporins were associated with a low incidence of CDI in the multivariate analysis, whereas the third- and fourth-generation cephaslosporins, penicillins, glycopeptides, carbapenems, and ketolides were statistically significant risk factors in the univariate analysis alone. This may be due to the fact that, clinically, many patients are treated with more than one type of antibiotic perioperatively for broad coverage.

A previous study reported that more than 10 days of antibiotics were identified as a significant risk factor for CDI^12^; however, although higher CDI incidence was noted when antibiotics were administered for a longer period of time, the trend was not statistically significant in our study. The period of antibiotics use was calculated as the number of days the antibiotics administered regardless of the number of antibiotics used. However, antibiotic days was defined as the summation of the number of days of administration of each antibiotic in the previous study^12^. The difference in the definition of the duration of antibiotic administration may have resulted in such disparity.

Multiple studies have reported on the risk of perioperative antibiotic prophylaxis and CDI. Prophylactic monotherapy with cefoxitin (second generation cephalosporin) was associated with a higher risk of CDI, and the addition of another antibacterial to cefoxitin further increased the risk^13^. The use of multiple antibiotics rather than the duration of antibiotic treatment was associated with CDI^14^. In the present study, the increased number of antibiotics was associated with an increased risk of CDI after spinal surgery. The Surgical Infection Society Guidelines on surgical prophylaxis recommend the use of antimicrobial prophylaxis for spinal procedures with or without instrumentation^15^. They also recommend monotherapy with cefazolin, either used as a single dose or administered up to 24 hours postoperatively^15^. Clindamycin and vancomycin could be used instead for patients with beta-lactam allergies. None of these antibiotics was associated with an increased risk of CDI in the present study, and therefore, these antibiotics could safely be used for prevention of both surgical site infection and CDI.

Although the surgical antibiotic prophylaxis recommends monotherapy with first-generation cephalosporin, other types of antibiotics or multiple drugs were prescribed perioperatively. We identified that prophylactic antibiotics were prescribed in combination in 21% of patients on the day of surgery. Among patients without any postoperative infection, only 30.5% was administered first-generation cephalosporins alone for surgical antibiotic prophylaxis. The second-generation cephalosporins were used as prophylactic antibiotics in many patients (15.3%), and many hospitals prescribed second-generation cephalosporins as discharge medication after intravenous administration of first-generation cephalosporins (18.2%). Similar pattern of such misuse of antibiotics was previously addressed in 2012 HIRA report on the adherence of surgical prophylaxis protocol. Combination of antibiotics was given to 19.8% of surgical patients, and 16.4% was given antibiotics as discharge medication^16^.

Similar to the results of the previously published papers on CDI after spinal surgery, we identified advanced age and postoperative infection, including urinary tract infection, as significant risk factors^6–8^. The difference from the previous studies is that we were able to identify antibiotic regimens and other perioperative medications. We demonstrated that the use of multiple antibiotics was a more significant risk factor than type of antibiotics and duration of use. There is a controversy regarding whether antacids increase the risk of CDI^17^. Although proton pump inhibitors were not shown to be associated with increased risk in the multivariate analysis, they were more commonly prescribed in the CDI patients. Therefore, caution should be taken when prescribing proton pump inhibitors to patients with an increased risk of CDI.

A novel finding in this study was that staged operations were shown to be an independent risk factor for CDI. Staged operation accounted for 0.2% of the cases, and it was associated with 5.336 times higher risk of CDI. Recently, the number of adult spinal deformity surgeries is rapidly increasing, and circumferential spine fusion is performed frequently^18,19^. Because these procedures are complex and the prolonged operation time poses significant burden on the patients, the surgeries are often performed in a staged manner^20^. Therefore, patients who receive staged operations may need more meticulous patient care and monitoring for development of postoperative complications, including CDI. Also, unlike in previous reports on CDI after spinal surgery, we subdivided postoperative infection into any infection, urinary tract infection, sepsis and pneumonia, and confirmed that all types of postoperative infection were risk factors for CDI.

Many studies have reported that CDI increases the length of hospitalization, medical cost, and mortality^6,21^. Similarly, we found that CDI increased mortality rate after spinal surgery by more than seven-fold, and CDI was associated with increased hospital stay and escalated treatment costs. The global surge in the incidence of hypervirulent *C. difficile* 027/BI/NAP1 subtype is likely to produce a significant burden on the health care system in the near future^13,22^.

The incidence of CDI after spinal surgery has been reported as 0.08–0.11%^6–8^. Our study found that the CDI incidence within 30 days after spinal surgery was 0.08%, which was lower than previous results. This disparity may be due to the difference in the definition of CDI. We identified CDI cases as those that satisfied both criteria of a specific diagnostic code and specific antibiotic administration, whereas other studies identified patients with the diagnoses alone^6,8^, or selected patients based on their medical record^7^.

There are several limitations in our study. We used administrative data and were unable to review the medical records for confirmation of the diagnosis or the treatment. The insurance claim data did not include individual health and nutritional status, such as body mass index, functional status, or smoking status, and it also did not provide laboratory data, such as hypoalbuminemia. Therefore, the severity or the stage of the conditions could not be investigated. Another inherent limitation of HIRA data is that there could be discordance between the diagnoses entered in the system and the actual health status of the patient, especially for mild conditions. Therefore, we could not calculate the comorbidity index and analyze its association with the CDI incidence. HIRA-NIS data includes insurance claim data of 13% of randomly selected inpatients, and each sample is labeled with an allocation number. The sample is renewed annually, and since the data is anonymous, a single patient who received operations more than once in different years may be classified as multiple different samples. Although we excluded surgery due to infection, we did not distinguish between trauma, degenerative disease, and congenital deformity. We also could not identify emergency surgeries. Furthermore, even though this study investigated 71,322 patients undergoing spinal surgery, the absolute number of patients (n = 57) with CDI was still low. Finally, our study restricted CDIs to those that occurred within 30 days after spinal surgery. Although many previous studies also used the same definition^6–8^, CDI has also been reported to occur after more than 30 days after antibiotics administration^23^.

In conclusion, we found that advanced age, staged operation, postoperative infection, and the use of multiple antibiotics were independent risk factors for CDI after spinal surgery. Although the incidence was low (0.08%), CDI was associated with negative outcomes of increased hospital stay, increased medical cost, and increased mortality. Therefore, modifiable risk factors should be minimized in order to prevent CDI and to optimize surgical outcome after spinal surgery. In particular, the unnecessary use of multiple antibiotics should be avoided.

## Methods

### Database and data collection

The National Health Insurance (NIH) covers about 98% of the South Korean population^24^. The data of the Health Insurance Review and Assessment Service (HIRA) includes information on patient diagnoses, past medical or surgical history, treatment procedures, and prescription dispensing information. We used the National Inpatient Sample of the HIRA (HIRA-NIS), which contains claims data of 13% of randomly selected inpatients. Data of patients who underwent spinal surgery from 2012 to 2016 were collected; those with spinal infections were excluded. Of the 71,761 patients, 293 patients were excluded due to spine-related infections and 146 patients were excluded from other types of infections.

The procedural codes of spinal surgery used in this study are described in Table 6. Outpatient based procedures such as vertebroplasty, kyphoplasty, neuroplasty and nerve block were excluded.

**Table 6:** Korea Informative Classification of Diseases procedural codes of spinal surgeries included in the study.

Procedural codes	
N1491	Diskectomy-Cervical Spine
N1492	Diskectomy-Thoracic Spine
N1493	Diskectomy-Lumbar Spine
N2491, N2492	Cervical Spine Laminoplasty
N1497, N2497	Laminectomy, Cervical Spine
N1498, N2498	Laminectomy, Thoracic Spine
N1499, N2499	Laminectomy, Lumbar Spine
N0466, N1466	Arthrodesis of Spine-Lumbar Spine-Anterior Technique
N0468, N1469	Arthrodesis of Spine-Thoracic Spine-Posterior Technique
N0469	Arthrodesis of Spine-Lumbar Spine-Posterior Technique
N1460, N2470	Posterior Lumbar Interbody Fusion
N2461, N2463	Arthrodesis of Spine-Cervical Spine-Anterior Technique
N2464, N2465, N2466	Arthrodesis of Spine-Thoracic Spine-Anterior Technique
N2467, N2468, N2469	Arthrodesis of Spine-Cervical Spine-Posterior Technique
N0451	Vertebral Corpectomy (Cervical Spine)
N0452	Vertebral Corpectomy (Thoracic Spine)
N0453	Vertebral Corpectomy (Lumbar Spine)
N0444, N0445	Arthrodesis for Spinal Deformity (Anterior Technique)
N0446, N0447	Arthrodesis for Spinal Deformity (Posterior Technique)
N0303	Osteotomy (Spine, Pelvis)

Presumed surgery-related CDI patients were identified as those diagnosed with enterocolitis due to *C. difficile* (International Classification of Diseases, Tenth Revision [ICD-10], diagnosis code A04.7.) and required administration of drugs used for CDI, such as metronidazole or vancomycin, within 30 days of spinal surgery.

Because HIRA-NIS data is a random sample and the sample population is renewed every year, we excluded patients who had surgery in December of each year for whom 30-day follow-up was not possible. According to the data from 2001 to 2010 in the United States, the incidence of CDI was highest in spring and lowest in autumn; therefore, excluding the data for December was unlikely to have a significant impact on the outcomes of our study^25^.

Patients were categorized into two groups: *C. difficile* infected and non-infected. Risk factors, such as age, sex, comorbidities, postoperative infection, spinal surgery procedure, staged operation, the types of antibiotics, and the duration of antibiotic use, were evaluated. Patients were presumed to have received staged operation if they received operation of the same site on different days, no more than 2 weeks apart, of the same hospitalization. The duration of the hospital stay, medical cost, and mortality were also evaluated.

This study was approved by the Institutional Review Board of Severance Hospital. Informed consent of the patients was waived since the study involved a retrospective access to publicly available and anonymized data. All methods were carried out in accordance with relevant guidelines and regulations.

### Statistical Analysis

Statistical analysis was performed by independent two-sample t-test and chi-square (or Fisher’s exact) test. For continuous variables, the data are expressed as a mean ± standard deviation. For categorical variables, the data are expressed as counts and percentages. To assess potential risk factors for CDI after spinal surgery, a multivariable logistic regression model was created to assess for multivariable predictors of CDI after spinal surgery. A multivariable logistic regression model was also constructed for mortality. Results are reported as odds ratio (OR) and 95% CIs. Risk factors associated with CDI diagnosis were assessed using multivariable logistic regression including all variables. The Hosmer-Lemeshow and model Χ2 goodness of fit tests were used for the model. All statistical analyses were performed with IBM SPSS Statistics for Windows/Macintosh, Version 23.0 (IBM Corp., Armonk, NY, USA). *P* values < 0.05 were considered significant.

## Data Availability

The data will be available upon reasonable request.
